# Dynamic and Seasonal Distribution of Enteric Viruses in Surface and Well Water in Riyadh (Saudi Arabia)

**DOI:** 10.3390/pathogens12121405

**Published:** 2023-11-29

**Authors:** Islem Abid, Albert Blanco, Nawal Al-Otaibi, Susana Guix, Maria I. Costafreda, Rosa M. Pintó, Albert Bosch

**Affiliations:** 1 Center of Excellence in Biotechnology Research, College of Applied Medical Science, King Saud University, Riyadh 11495, Saudi Arabia; iabid@ksu.edu.sa; 2 Enteric Virus Laboratory, Department of Genetics, Microbiology and Statistics, School of Biology, University of Barcelona, 08028 Barcelona, Spain; ablancoortiz@ub.edu (A.B.); susanaguix@ub.edu (S.G.); mcostafreda@ub.edu (M.I.C.); rpinto@ub.edu (R.M.P.); 3 Research Institute of Nutrition and Food Safety (INSA-UB), University of Barcelona, 08921 Barcelona, Spain; 4 Department of Botany and Microbiology, Science College, King Saud University, Riyadh 11495, Saudi Arabia; nawalso@hotmail.com

**Keywords:** hepatitis A, hepatitis E, norovirus, real-time RT-qPCR

## Abstract

Enteric viruses are the major cause of gastroenteritis and enteric hepatitis worldwide, but in some areas like Saudi Arabia, little is known about their presence in water sources. The available information from clinical samples is not enough to figure out their actual prevalence. The aim of this study was to gather information for the first time in Saudi Arabia on the presence of the Norovirus (NoV) genogroup GI and GII, hepatitis A virus (HAV), and hepatitis E virus (HEV) in water. For this purpose, thirteen monthly samples were collected from Lake Wadi Hanifa and surrounding wells from December 2014 to November 2015. Viruses were detected and quantified using real-time RT-qPCR. Despite HEV findings being anecdotic, our results highlight interesting behaviors of the other viruses. There was a higher prevalence of noroviruses in Wadi Hanifa samples than in well water samples (46.43% vs. 12.5% of NoV GI; 66.67% vs. 8.33% of NoV GII). On the contrary, similar levels of HAV positivity were observed (40.48% in surface water vs. 43.06% in well water). Also, a strong influence of flooding events on HAV and NoV GI occurrence was observed in both surface and well water samples, with NoV GII apparently not affected.

## 1. Introduction

Enteric viruses, the major cause of acute gastroenteritis and enteric hepatitis worldwide, are among the most important waterborne pathogens [1]. Despite their divergence in pathogenesis and life cycle, they are all transmitted by the fecal-oral route through both food and water contamination. They spread in the environment mostly as non-enveloped particles, making them resistant to unfavorable conditions and persisting for extended periods. Their infectious dose is low [2], and combined with their extremely high excretion of up to 10^8^–10^11^ genome copies/gr of feces [3], enteric viruses are capable of producing large outbreaks. The most important viruses in this group can be divided into enterotropic and hepatotropic viruses, i.e., norovirus (NoV) and hepatitis A virus (HAV), the best known and characterized of each category [4].

NoV is the causal agent of approximately 20% of all gastroenteritis, infecting 685 million people every year and causing 200,000 deaths per year, making them the leading cause of gastroenteritis in the United States. However, it also has a high prevalence of up to 21% in regions such as Latin America, North Africa, the Middle East, China, and low-income countries [5,6,7]. Genus *Norovirus*, within the *Caliciviridae* family, has high diversity, and all known NoV are classified into 10 different genogroups (GI to GX), but only NoV GI, GII, GIV, GVIII, and GIX are capable of infecting humans, with GI and GII being the most common. NoV GI is less commonly found in patients with acute gastroenteritis across the globe than NoV GII [8,9,10]. Genotype GII.17 emerged in Asia and was responsible for gastroenteritis linked with NoV in 2014, which has subsequently been documented worldwide [11,12]. Across the world, NoV has been detected in different water bodies, including rivers, sewage, municipal water, groundwater, and recreational water [13,14]. NoV can cause both sporadic cases and outbreaks all year, with a significant seasonality, having a peak of incidence in cold seasons [15].

HAV is a member of the *Hepatovirus* genus, belonging to the family *Picornaviridae*, and causes acute viral hepatitis. Based on the capsid nucleotide variability, three genotypes (I, II, and III) and six subgenotypes (IA, IB, IIA, IIB, IIIA, and IIIB) of HAV have been described [16]. The primary mechanism of transmission of HAV is the fecal-oral route and direct contact with an infected person [17]. Thus, the virus is present in different water environments, and it is extremely resistant to adverse conditions: it can stay infectious for about 60 days in tap water, more than 6 weeks in river water, over 8 weeks in groundwater, and about 30 weeks in seawater [18,19]. One and a half million people suffer from hepatitis A annually, which is an underestimation of the number of infected people due to the asymptomatic presentation of the virus [4]. Hepatitis A outbreaks are mainly associated with water supplies [20], but foodborne transmission [19,21,22] and infections between men having sex with men are also common [23]. The consumption of improperly cooked or raw oysters and clams from sewage-contaminated water has led to multiple outbreaks of HAV infection [24]. Hepatitis A can be prevented by vaccination, but the vaccine is not administered worldwide. The global distribution of hepatitis A is divided into non-endemic countries and endemic countries. Non-endemic regions are usually high-income countries with improved hygienic-sanitary conditions, whereas endemic regions typically correspond to low-income countries with poor water sanitation [16].

In recent years, hepatitis E virus (HEV), a viral agent with zoonotic potential, has emerged. HEV causes foodborne hepatitis, sometimes with chronicity in immunocompromised individuals [24], and complications in pregnant women that may even lead to a high mortality rate [25,26]. HEV virus is a foodborne and waterborne pathogen threatening global health in developed and developing countries [27,28]. HEV has eight different genotypes: genotypes 1–4 and 7 are known to be the main threats to humans [29]. Genotype 7 (HEV-7) was first isolated in camels [30,31]. Another genotype related to camels was also described (HEV-8), but it has yet to be demonstrated that it can infect humans [32].

Riyadh city, the capital of the kingdom of Saudi Arabia, has an arid environment with minimal rainfall and extreme temperatures in the summer months. For this reason, water is a rare and precious commodity. The scarcity of this resource has increased due to the explosive demographic increase in the area, moving from 150,000 inhabitants to about 6.5 million in sixty years [33]. In addition, the government is covering the big demand for water for agriculture by using two conventional sources and a non-conventional one. Conventional resources include surface water and groundwater, whereas the non-conventional source is treated wastewater. Twenty-five percent of tertiary treated wastewater is used to irrigate landscapes in public parks in a number of cities and crop-irrigated areas across Saudi Arabia. Wadi Hanifa Lake, which is the city’s main drainage system and is used for irrigation, receives treated wastewater from the Manfuha sewage treatment plant [34].

Several enteric pathogens, including Astrovirus, Rotavirus A, and Adenoviruses, have been detected in stool samples of children with gastroenteritis in Saudi Arabia and in water environments using simple molecular detection techniques [35,36,37,38,39,40]. Taking into account that Saudi Arabia is an intermediate region regarding HAV endemicity, with a high risk of HEV because of close contact with camels, the virology profile of both Wadi Hanifa Lake and well water may provide valuable information on the prevalence and survival of enteric viruses in desert environments. In the present study, we investigated for the first time the occurrence of NoV GI and GII, HAV, and HEV in water samples of Wadi Hanifa Lake and the neighboring wells through RT-qPCR.

## 2. Materials and Methods

### 2.1. Sample Collection

**Figure 1:**
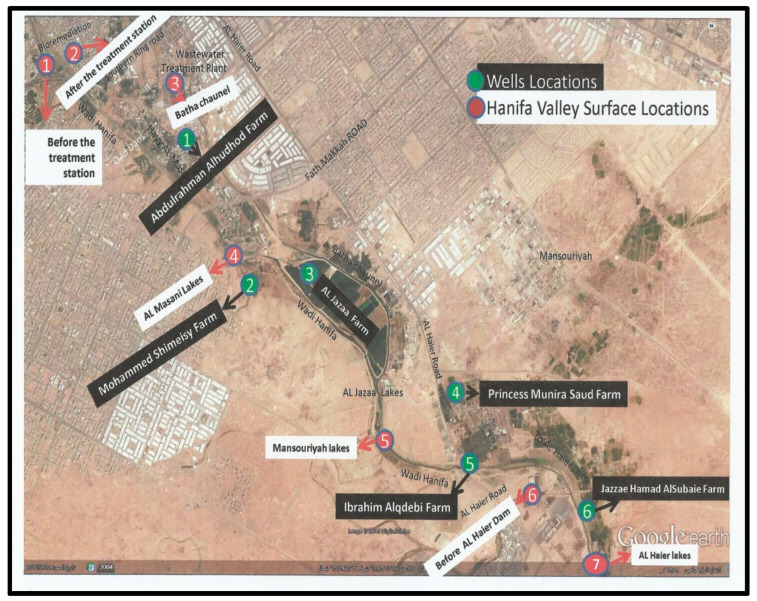
Sampling locations in Google Maps (red: surface water (S1–S7); green: well water (W1–W6)).

Samples (10 L) were collected monthly from December 2014 to November 2015 to evaluate the presence of enteric viruses in different water environments in Riyadh and ascertain the prevalence and seasonality of viruses. Locations S1–S7 were selected strategically to cover most of the Wadi Hanifa Lake surface area, and locations W1–W6 covered the wells between the Wadi Hanifa and Batha channels (Figure 1). The location of each sampling site was as follows: S1, before the raw water treatment in the bioremediation treatment plant; S2, after the water treatment in the bioremediation treatment plant; S3, Batha stream channel after leaving the Manfuha treatment plant; S4, waterway after lake factories directly; S5, waterway after Mansoriyah lake; S6, watercourse before Dam Al Haierlake; S7, watercourse after Dam Al Haier lake. The bioremediation treatment plan uses the foodchain for the treatment of urban wastewater by combining both primary producers and consumer organisms with the support of a low-tech eco-centric infrastructure that maintains the ecosystem. This treated water is used for urban functions such as a river park system and increase the water flow in Wadi Hanifa. The choice of wells was dependent on their proximity to Wadi Hanifa Lake and the permission of their owners.

The total number of samples was 156 (84 surface water samples from Wadi Hanifa and 72 well water samples). Samples were collected in sterile containers and transported on ice to the laboratory, where they were kept at 4 °C until processing within 24 h. Physicochemical and microbiological analyses were performed in all samples prior to the concentration. Furthermore, temperatures were obtained and recorded on each collection day, from the AccuWeather website. Table S1 shows the average water temperatures of the surface water samples.

### 2.2. Viral Concentration

All water samples were concentrated using an optimized glass wool filtration method [41] in the Department of Botany and Microbiology of the King Saud University. Briefly, each water sample passed through a positive-charged glass wool filter to detain all viruses in the sample. Then, the viruses were eluted with 200 mL of glycine beef extract (GBE) buffer (glycine 0.05 M, beef extract 3%) at pH 11. The buffer was recirculated through the filter for 1 h to improve the recovery of enveloped viruses. After this elution, 20% of polyethylene glycol (PEG) 6000 was added to the eluate, and a secondary concentration based on flocculation precipitation was performed. The concentrate (2 mL) was stored at −80 °C until the nucleic acid extraction.

### 2.3. Total Nucleic Acid Extraction

Total RNA was extracted from 500 μL of sample using the NucliSENS^®^ miniMAG^®^ extraction system (Biomérieux, Marcy-l’Étoile, France). Nucleic acid extraction was performed according to the instructions provided by the manufacturer, obtaining a final volume of 100 μL. To evaluate nucleic acid extraction efficiency, 10 μL of Mengovirus strain MC_0_ was added to each sample before the lysis step [42,43]. Once extracted, all samples were stored at −80 °C.

### 2.4. Virus Detection and Quantification (RT-qPCR)

#### 2.4.1. HAV, NoV GI, and NoV GII

Mengovirus quantification and HAV, NoV GI, and NoV GII screening were performed using a multiplex real-time RT-qPCR as previously described [42]. Mengovirus recovery efficiencies ≥1% were considered acceptable, whereas recoveries <1% were considered unacceptable, and the nucleic acid extraction was repeated. Once acceptable efficiencies were reached for all samples, they were tested for the presence of HAV, NoV GI, and NoV GII. Positive samples were then processed in a monoplex RT-qPCR for virus quantification. The monoplex RT-qPCR was selected for quantification because the multiplex assay is slightly less sensitive. As described in [42], the theoretical limits of detection with 95% confidence were 491, 23, and 33 copies per reaction in the multiplex assay for HAV, NoV GI, and NoV GII, respectively, and 51, 2, and 17 copies per reaction, respectively, in the monoplex assay. Positive samples that were not detected in the monoplex or those that were below the limit of quantification were qualified as “detectable non-quantificable” samples and were arbitrarily scored as bearing <5 genome copies/reaction (rxn) since the different targets showed different LoQ.

Both multiplex and monoplex RT-qPCR have the same cycling parameters: 60 min at 55 °C for reverse transcription and 5 min at 95 °C for initial denaturalization, followed by 45 cycles consisting of 15 s at 95 °C, 60 s at 60 °C, and 60 s at 65 °C for amplification. Fluorescence was read at every cycle after the last step [43,44,45,46].

#### 2.4.2. HEV

HEV was detected and quantified by real-time RT-qPCR using the primers and probes previously described [20]. The amplification program was modified as follows: 30 min at 50 °C for reverse transcription and 10 min at 95 °C for initial denaturalization, followed by 45 cycles consisting of 15 s at 95 °C and 60 s at 58 °C for amplification. Fluorescence was read at every cycle. All RT-qPCRs were performed using the Ultrasense One Step RT-qPCR System kit, (Thermofisher^®^, Cornellà de Llobregat, Spain), and Stratagene^®^ Mx3000p thermocycler (Agilent Technologies Inc., Santa Clara, CA, USA). All sets of primers and probes are shown in Table 1.

### 2.5. Statistical Analysis

Neither prevalence nor quantification data were normally distributed; differences in positivity and comparison between mean viral loads were performed using the Mann–Whitney test. *p* values < 0.05 were considered statistically significant. All tests were performed using SigmaPlot 11.0.

## 3. Results

### 3.1. Seasonal Distribution of Enteric Viruses in Surface Water

#### 3.1.1. Prevalence of Hepatitis Viruses

**Figure 2:**
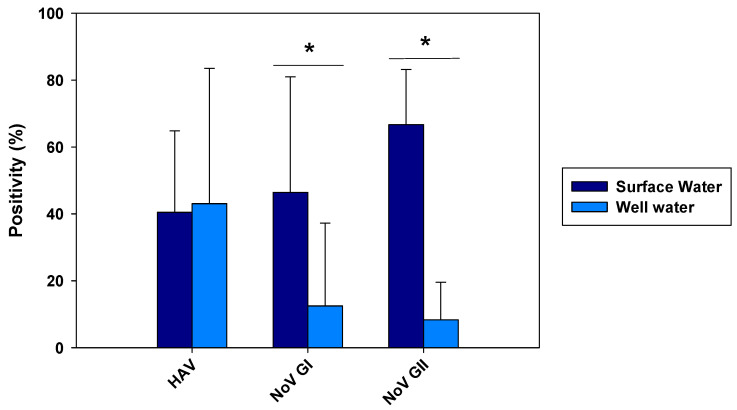
Prevalence of HAV, NoV GI, and NoV GII in surface and well water samples. The data shown are the mean and SD of monthly positivity percentages. Differences between surface and well water samples were assessed using the *t*-test (* *p* < 0.05).

**Figure 3:**
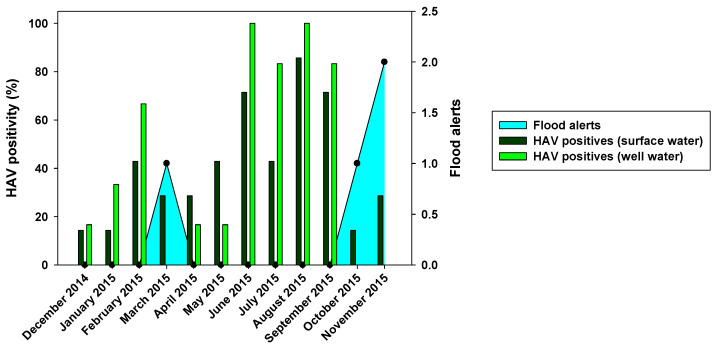
Monthly HAV positivity for both surface and well water compared with flood alerts. Black dots depict the occurrence of floods.

From all 84 samples analyzed from the Wadi Hanifa surface water, 34 (40.48%) were positive for HAV (Figure 2). HEV was not detected in any surface water sample. Regarding the seasonality of HAV, the result of this study revealed that it was present in the surface water of Wadi Hanifa Lake all year round. The highest HAV prevalence (or positivity) was found during summertime (June–September), with August being the month with the highest prevalence (83.3%), whereas the lowest prevalence (15%) was observed during the winter season (December–January), (Figure 3).

#### 3.1.2. Prevalence of NoVGI and GII

**Figure 4:**
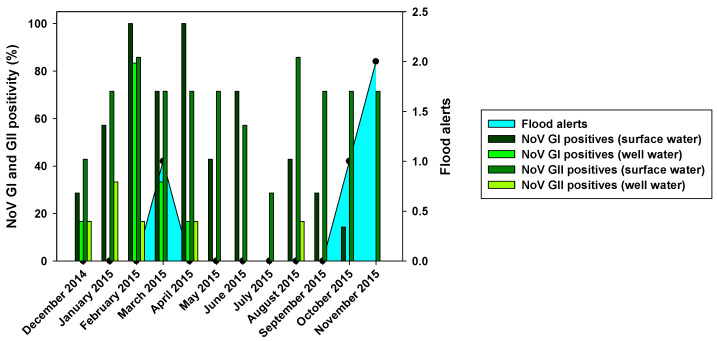
Monthly NoV GI and GII positivity for surface and well water related to flood alerts. Black dots depict the occurrence of floods.

The prevalence of NoV was also very high. In fact, from the 84 samples analyzed for NoV GI and GII, 39 (46.43%) were positive for NoV GI, and 56 (66.67%) were positive for NoV GII (Figure 2). NoV GI was present all year round with the exception of two months: July and November (Figure 4). NoV GI prevalence was high from February to June, having their highest in February and April. On the other hand, the NoV GII prevalence was mostly stable throughout the year, varying between 30 and 85%.

### 3.2. Seasonal Distribution of Enteric Viruses in Wells

#### 3.2.1. Prevalence of Hepatitis Viruses

A total of 72 samples were analyzed from neighboring wells of Wadi Hanifa Lake for HAV and HEV; 31 samples (43.06%) were positive for HAV, whereas only two (2.77%) were positive for HEV. One of them was collected in May in the fourth well water sample location (W4), and the other in November in the first well water sample location (W1).

HAV was present mostly all year round, but it was extremely prevalent in the summer season (95%), while the lowest presence was observed in the cold season (0–15%) (Figure 3). These results are in line with those found in the surface water. We can observe a relationship between the abundance of HAV in Wadi Hanifa Lake and its high prevalence in wells.

#### 3.2.2. Prevalence of NoV GI and GII

Out of 72 well water samples tested for NoV, 9 (12.5%) were positive for NoV GI and 6 (8.33%) for NoV GII (Figure 2). The highest virus prevalence was in the wet season, which includes the winter and spring months, with an additional finding of GII in August (Figure 4). Despite a reduction in viral loads, probably due to the viral loss in water filtration from surface water to wells, we could observe that the months with the highest NoV prevalence in surface water were also the ones with the highest positivity in well water.

### 3.3. Differences between Sampling Areas

Two of the most interesting sampling areas in this study were S1 and S2 locations. S1 is located before the bioremediation station that uses the biological trophic chain in nature to increase Wadi Hanifa’s water quality [47]; S2 is located in an area that receives treated water from this bioremediation station. Sampling areas S3 to S7 are located further away from the Manfuha wastewater treatment plant (Figure 1).

**Figure 5:**
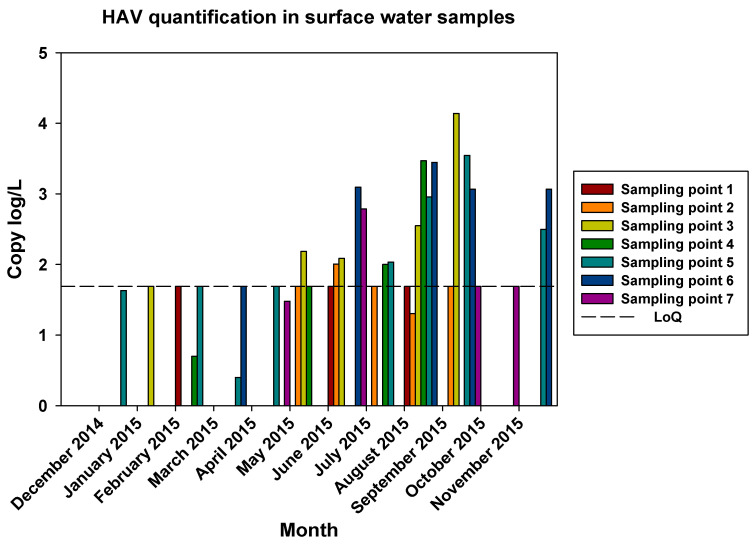
Monthly viral load of HAV detected in all seven surface water sampling points.

**Figure 6:**
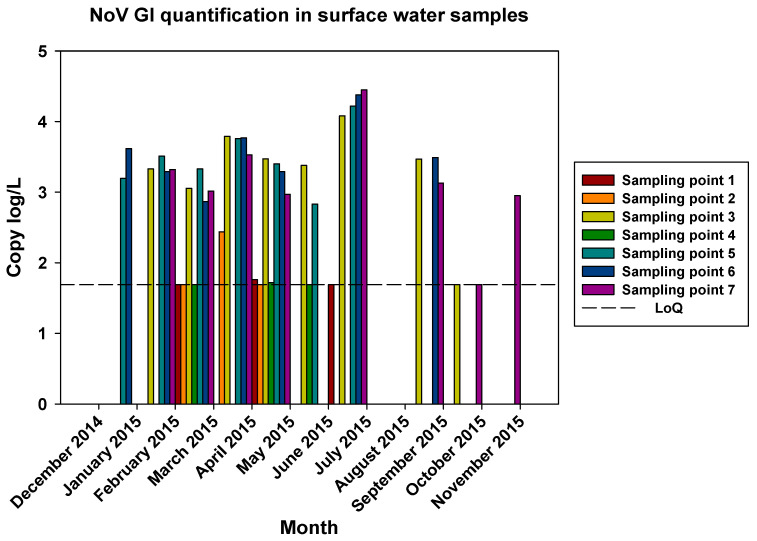
Monthly NoV GI viral load in all seven surface water sampling locations.

**Figure 7:**
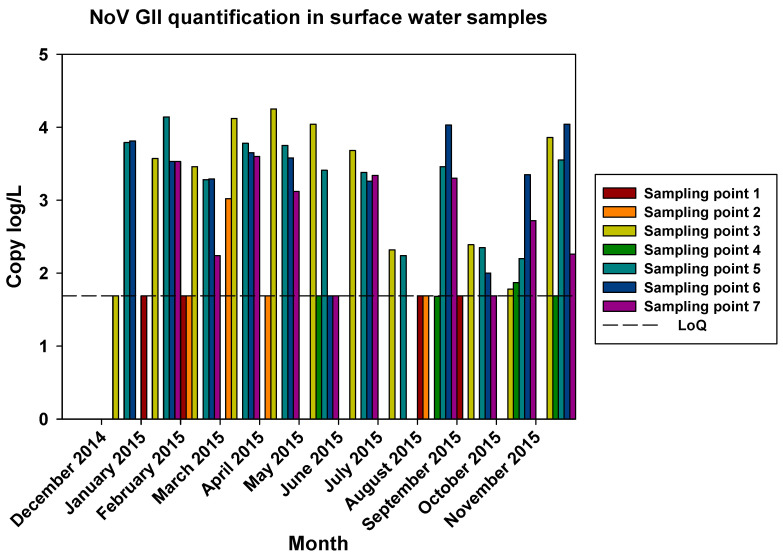
Monthly NoV GII viral load in all seven surface water sampling locations.

The comparisons of the viral loads of all three viruses at the different sampling points over time are represented in Figure 5, Figure 6 and Figure 7. By comparing the viral loads between S1 and S2 samples, we observed that Wadi Hanifa’s bioremediation station could neither eliminate nor significantly reduce the presence of enteric viruses in water. In some months, HAV, NoV GI, or NoV GII were detected in both S1 and S2 samples (Figure 5 and Figure 6). We could also detect HAV more frequently in the S2 sampling location than in the S1 location (Figure 5).

The prevalence of all three viruses tended to increase as samples were taken further away from the Manfuha wastewater treatment plant. In S5, S6, and S7 locations, we could detect the maximum number of positive samples (50% to 100% positivity, depending on the virus), and we could also detect the higher concentration of genomic copies in these samples. We detected the maximum prevalence in the S5 location for HAV and NoV GII and in S7 for NoV GI. The S6 location had the highest mean viral load for all three viruses: 2.68 log of genome copies/L (log gc/L) for HAV, 3.55 log gc/L for NoV GI, and 3.35 log gc/L for NoV GII.

The S3 sampling location had a significantly high prevalence of NoV GI (with the same prevalence as S7) and NoV GII compared to the S1 and S2 locations, but not for HAV. This latter one was an interesting sampling location because we could also detect one of the highest concentrations for all three viruses despite it being located immediately after the treatment plant. The mean viral loads of the positive samples collected in this location were 2.53, 3.28, and 3.23 log gc/L for HAV, NoV GI, and NoV GII, respectively.

### 3.4. Comparison of Viral Load in Wadi Hanifa Lake and the Neighboring Wells

The positivity of Wadi Hanifa Lake samples and well water samples were recorded and compared in Figure 2. The results showed that the prevalence of both groups of NoV was significantly higher in the lake than in the wells (*p* < 0.05). Regarding HAV, there was no significant difference between the surface water and well water. Considering the samples in each season separately (May–September for the dry season and October–March for the wet season), no significant differences were observed between surface and well water samples for HAV in any season (Table 2). In contrast, significant differences in the viral loads of NoV GI and NoV GII were detected between the surface and well water during the dry season. Concerning HEV which was found only in two different wells, the viral load was 2.96 log gc/L and 2.71 log gc/L in May and November, respectively.

When quantifying positive samples, the mean log gc/L ranged between 1.04 and 2.83 for HAV in Wadi Hanifa Lake and between 1.66 and 2.72 in wells. The highest mean values were observed in September, whereas the lowest mean values were in March. According to these data, we can confirm that HAV levels in the wells increased in the hot season (Table 2). Regarding NoV, the mean log of gc/L ranged between 1.69 and 3.76 for NoV GI and between 1.96 and 3.63 for NoV GII in the surface water of Wadi Hanifa Lake. The highest mean values were observed in June and March for NoV GI and NoV GII, respectively. The lowest mean values were in September and July for NoV GI and NoV GII, respectively.

## 4. Discussion

Wadi Hanifa Lake runs through the city of Riyadh, and around 70% of the city is located within its catchment area. The term “Wadi” usually refers to a dry riverbed that contains water only after heavy rain episodes, and this is the natural condition of Wadi Hanifa. However, the sampling area covered in the present study, in the south of Wadi Hanifa, bears a continuous flow of water resulting from the effluents of the Manfuha sewage treatment plant, drained Riyadh groundwater, and stormwater channels draining different sectors of the city [48]. It also represents a convenient system for disposing of Riyadh wastewater.

Temperatures in summer reach an average of 42.9 °C (109.2 °F), and precipitation averages only 60 mm (2.4 in) per year in the driest places. Rain falls with great intensity for short periods between October and April, causing flash floods. Usually, April is the rainiest month, but 2015 had atypical heavy rains with floods in October and November, affecting the results. The nature of the dry, warm climate leads to a high percentage of the scarce rainfall being instantly evaporated. The remaining water mostly ends up as groundwater. While abundant, the levels of the water table are being tested by the rapid growth the city of Riyadh has seen in the past fifty years, from a population of 150,000 in 1960 to an estimated 6.5 million today. For that, Wadi Hanifa surface and well water are of particular interest because of their potential to contain high titers of enteric viruses.

The prevalence of NoV GI and NoV GII, as summarized in Figure 2, showed differences in positivity between surface and well water samples, which was probably due to water filtration processes. The similar HAV positivity for both types of samples and lower NoV prevalence in well water samples would mean that HAV is more resistant to water filtration than NoV. The higher HAV prevalence in both well water and surface water was detected in the driest months (June–August). That could point out that groundwater protects viruses from the temperature, sunlight, and drought. For NoV, most of the positive samples were detected in December–April, and then prevalence decreased for NoV GI, with minimum prevalence in the first two months of the wet season. HAV shows similar behavior toNoV GI, but its higher prevalence was detected in June–September. For well water samples, all three viruses were absent in rainy months. Heavy rain and floods could dilute viruses in water, causing NoV GI and HAV in surface water, and all three viruses in the well water were harder to detect. The prevalence of NoV GII seems to be unaffected by rain since its positivity was about the same before and after the rainy period. There was also a negative correlation between rainfall and HAV and NoV GI prevalence. For these two viruses, all three months with floods registered (March, October, and November) showed significant decreases in prevalence in surface and well water (Figure 3 and Figure 4). Despite that, the mean viral load of HAV in November was one of the highest found in the study. Previous studies showed that since the vaccination program started in 2008, the seasonality of HAV has begun to shift from detecting most of the hepatitis A cases in summer to being able to detect hepatitis A cases in autumn [33]. Our data corroborates this seasonality shift since the highest viral loads are detected not only in November but also in the second half of the summer months (August and September). No correlation between NoV GII and rainfall was observed. Nevertheless, in March 2015, NoV GII was not detected in well water samples (Figure 4).

Surface water presents a certain HAV-NoV duality when comparing viral loads. HAV had its highest titer in August and September 2015 for well water and surface water, respectively. In these months, NoV GI had its minimum viral load in surface water samples, and NoV GII quantification was lowest in surface water and minimum in well water as well. Additionally, lower HAV viral loads correlated with higher viral load periods for NoV (January–March 2015). Similar results were found in the well water samples but only for NoV GI and HAV since NoV GII was mostly undetectable.

In Saudi Arabia, there is little published evidence on HAV incidence, unlike its seroprevalence. In 2021, the seroprevalence of HAV oscillated between 8% and 100%, with the highest incidence reported in the Eastern region compared to the Central and Western regions [31]. In this study, a sustained shift in hepatitis A endemicity compared with what was recorded before the implementation of the vaccine was observed. In our study, we found a high prevalence of HAV (approximately 40%) between December 2014 and November 2015. This result was completely unexpected, considering the fact that Saudi Arabia implemented a childhood HAV vaccination program in 2008, and Badur et al. in 2021 reported that the total number of new hepatitis cases declined by about 90% after 2008 [33]. The same study points to a significant increase in hepatitis A incidence in the years 2016 to 2018. The surprisingly high viral load in November 2015 could indicate the beginning of that episode of higher HAV incidence. Thanks to the hepatitis A vaccination program, the incidence of HAV in Saudi Arabia decreased from approximately 6.7 cases per 100,000 population in 2008 to less than 1 case per 100,000 population in 2014–2015, with a total number of reported cases of less than 250 in the country [33]. Since the mass vaccination against hepatitis A was initiated in 2008 in children at 18 months of age, and individuals ≥15 years of age constitute the majority of the total population (67.6%), most inhabitants are still susceptible to HAV infection. According to data collected in 2021, Saudi Arabia has a population of around 36 million, with 3–5 million illegal immigrants [33], with over 20% of the population living in Riyadh. Thus, the high population density and immigration in the capital, together with the large proportion of individuals who are still susceptible to HAV infection, may explain the high prevalence of HAV despite the effectiveness of the vaccination program.

HEV was detected in two well water samples; hence, it seems that there is no significant shedding of this virus in the environment. Few data were found regarding the prevalence of HEV in Saudi Arabia in humans. Arif et al. reported that the seroprevalence of HEV in Riyadh was 8.4% [35]. More recently, Al Dossary et al. in 2021 reported that the seroprevalence of HEV in the Eastern Province of Saudi Arabia was low (3.2%) [49]. However, recent serological studies in camels showed a prevalence of 23.1% in local camels in Jeddah province [50]. This high prevalence in camels highlighted the role of camels as a zoonotic reservoir for HEV infection in humans. Further investigations are needed to know the actual prevalence of HEV in humans in Riyadh and Saudi Arabia. On the other hand, the absence of HEV-positive samples could be due to the employed RT-qPCR assay that only detects the major HEV genotypes (HEV-1 to HEV-4) and not camel HEV genotypes. In fact, a novel HEV genotype, named Dromedary camel HEV, which was first detected in 2014 in camels in the Middle East, was reported to be the main cause of acute hepatitis in a transplant patient in the United Arab Emirates who regularly consumed camel milk and meat [51]. In Saudi Arabia, more precisely in Jeddah province, the prevalence of HEV was low (1.77%) in domestic camels [52]. The absence of data in Riyadh prevented us from drawing definite conclusions.

Regarding NoV in Saudi Arabia, there is no information on its prevalence except for one report in the literature [37]. In this study, 1000 stool samples were collected and screened for many enteric viruses. The prevalence of NoV was determined by ELISA, and it was 3.5%. Our data in this study showed that the prevalence of NoV GI and GII in the surface water of Wadi Hanifa Lake was between 45% and 65%, which is higher than what was reported in the study of Tayeb et al. The use of a quantitative molecular technique, such as the real-time RT-qPCR, provided more precise data in our work. We conclude that the incidence of NoVin Saudi Arabia is so far underestimated. We hope that our results will provide the authorities with useful information to focus on these viruses and take appropriate measures. Improving wastewater treatment in several treatment plants in Riyadh could efficiently reduce the transmission of such viruses through lakes, which are used for irrigation and agricultural purposes.

In any case, this study has the limitation of covering only one year, and further research is needed to confirm our data.

## 5. Conclusions

Monitoring human viruses in environmental waters is critical to gather complementary information for the adoption of public health measures for the prevention of diseases, as well as for the implementation of mitigation measures in response to outbreaks. Hence, conducting regular viral monitoring of treated wastewater discharged into the environment is important for the prevention of diseases associated with exposure to virus-contaminated water. Meanwhile, the detection of viral genome copies in water and wastewater does not necessarily mean that the detected particles are infectious. However, genome copies are indicative of potential infections and health risks associated with the virus-contaminated matrices.

Since the information on the presence of enteric viruses in water in Saudi Arabia is scarce, it is hard to establish comparisons with other data, representing a limitation of the present study. Nevertheless, we can confirm the higher prevalence of HAV and NoV in surface water than in well water samples. Quantitative data showed that both NoV GI and GII are present in higher viral levels in Wadi Hanifa than in the surrounding wells, although this is not the case for HAV, showing similar titers in well and surface waters.

Our data also point to strong rainfall events that negatively correlate with HAV/NoV GI prevalence in surface water, although only with HAV in well water. We could also observe opposite patterns between the viral loads of HAV and both NoV GI and GII.

The sporadic detection of HEV points to the low prevalence of the major genotypes of this pathogen in Saudi Arabia’s environment. However, the assessment of the zoonotic potential of HEV-7 and the recent description of HEV-8, both of which have camels as their natural reservoir and show high seropositivity, make HEV screening a major priority in Saudi Arabia.

## Figures and Tables

**Table 1:** Primers and probes employed for virus detection and quantification. Nucleotide positions correspond to the sequences of the reference strains MC_0_ (Genbank L22089) for Mengovirus, HM175 (Genbank M14707) for HAV, Norwalk (Genbank M87661) for NoV GI, Lordsdale (Genbank X86557) for NoV GII and genotype 1a (GenBank M73218) for HEV.

Virus (Nt Position)	Primers
Mengovirus (110–209)	Fw: 5′-GCGGGTCCTGCCGAAAGT-3′
	Rv: 5′-TGCACGCCATCTTCATTCACA-3′
	Probe (Multiplex): 5′-[VIC]-AGCACGTGGGAGGGCGATCG-[MGB]-3′
	Probe (Monoplex): 5′-[6FAM]-AGCACGTGGGAGGGCGATCG-[MGB]-3′
Hepatitis A virus (68–240)	Fw: 5′-TCACCGCCGTTTGCCTAG-3′
	Rv: 5′-GAGCCCTGGAAGAAAG-3′
	Probe (Multiplex): 5′-[6FAM]-CCTGAACCTGCAGGAATTAA-[MGB]-3′
	Probe (Monoplex): 5′-[6FAM]-CCTGAACCTGCAGGAATTAA-[MGB]-3′
Norovirus GI (5291–5376)	Fw: 5′-CGCTGGATGCGNTTCCAT-3′
	Rv: 5′-CCTTAGACGCCATCATCATTTAC-3′
	Probe (Multiplex): 5′-[TxRED]-TGGACAGGAGAYCGCRATCT-[IBRQ]-3′
	Probe (Monoplex): 5′-[6FAM]-TGGACAGGAGAYCGCRATCT-[TAMRA]-3′
Norovirus GII (5012–5100)	Fw: 5′-ATGTTCAGRTGGATGAGRTTCTCWGA-3′
	Rv: 5′-TCGACGCCATCTTCATTCACA-3′
	Probe (Multiplex): 5′-[ATTO]-AGCACGTGGGAGGGCGATCG-[BHQ]-3′
	Probe (Monoplex): 5′-[6FAM]-AGCACGTGGGAGGGCGATCG-[TAMRA]-3′
Hepatitis E virus (5261–5330)	Fw: 5′-GGTGGTTTCTGGGGTGAC-3′
	Rv: 5′-AGGGGTTGGTTGGATGAA-3′
	Probe: 5′-[6FAM]-TGATTCTCAGCCCTTCGC-[BHQ]-3′

**Table 2:** Monthly HAV, NoV GI, and NoV GII viral loads in the surface and well water samples. NA means that none of the samples were positive for that virus.

	Viral Load (Log_10_ Mean ± SD)					
Month	Surface Water			Well Water		
	HAV	NoV GI	NoV GII	HAV	NoV GI	NoV GII
December 2014	1.63 ± 0.00	3.40 ± 0.21	3.10 ± 0.99	<1.69 ± 0.00	<1.69 ± 0.00	<1.69 ± 0.00
January 2015	<1.69 ± 0.00	3.36 ± 0.09	3.29 ± 0.83	<1.69 ± 0.00	NA	1.75 ± 0.05
February 2015	1.20 ± 0.50	3.06 ± 0.17	2.60 ± 0.75	<1.69 ± 0.00	1.94 ± 0.39	<1.69 ± 0.00
March 2015	1.04 ± 0.65	3.45 ± 0.52	3.63 ± 0.36	NA	1.78 ± 0.08	NA
April 2015	1.58 ± 0.11	2.46 ± 0.78	3.27 ± 0.87	<1.69 ± 0.00	<1.69 ± 0.00	<1.69 ± 0.00
May 2015	1.86 ± 0.23	2.63 ± 0.70	2.50 ± 1.01	<1.69 ± 0.00	NA	NA
June 2015	2.33 ± 0.52	3.76 ± 1.04	3.41 ± 0.16	2.15 ± 0.34	NA	NA
July 2015	1.91 ± 0.15	NA	1.96 ± 0.27	1.66 ± 0.34	NA	NA
August 2015	2.57 ± 0.83	3.36 ± 0.17	3.02 ± 0.97	2.72 ± 0.47	NA	1.68 ± 0.00
September 2015	2.83 ± 0.98	1.69 ± 0.00	2.02 ± 0.30	1.90 ± 0.17	NA	NA
October 2015	1.69 ± 0.00	2.94 ± 0.00	2.37 ± 0.58	NA	NA	NA
November 2015	2.78 ± 0.29	NA	3.08 ± 0.93	NA	NA	NA

## Data Availability

Data will be made available on request.

## Supplementary Materials

The following supporting information can be downloaded at: https://www.mdpi.com/article/10.3390/pathogens12121405/s1, Table S1: Average surface water temperature at the time of sample collection.
