# Supplementary material for: Dynamic and Seasonal Distribution of Enteric Viruses in Surface and Well Water in Riyadh (Saudi Arabia)
Source: Pathogens. 2023 Nov 29;12(12):1405. doi: 10.3390/pathogens12121405 (PMC10747075; doi:10.3390/pathogens12121405)
Supplement: Supplementary file 1 [file pathogens-12-01405-s001.zip › pathogens-2669839-supplementary.pdf]

Supplementary Materials

Table S1. Average surface water temperature at the time of sample collection.

Month	Average T (S1-S7) ℃
December	23.9
January	21.2
February	24.5
March	25.0
April	27.0
May	29.5
June	29.5
July	30.2
August	31.0
September	30.0
October	29.1
November	25.0
